# Percolation with Distance-Dependent Site Occupational Probabilities

**DOI:** 10.3390/e28010128

**Published:** 2026-01-22

**Authors:** Eleftherios Lambrou, Panos Argyrakis

**Affiliations:** Department of Physics and Complexity Center, University of Thessaloniki, GR-54124 Thessaloniki, Greece; lamele@pogoni.gr

**Keywords:** inverse percolation, distance-dependent occupational probability, hypoxia heterogeneity, tumor radiotherapy, Monte-Carlo simulations, oncology radiation modeling

## Abstract

We introduce a new method for preparing a percolation system by employing an inverse percolation model. Unlike standard percolation, where the site occupancy is uniform, the new model imposes a distance-dependent probability of site removal, where sites closer to the lattice center have a higher probability of being removed and are more prone to damage as compared to those at the periphery of the system. The variation in this removal probability is a function of the distance (d) from the central point. Thus, the central point plays a key role. This is reflected in our effort to model the role of a tumor cell and its surroundings (the tumor microenvironment). The tumor causes a decrease in the concentration of key elements, such as $O_{2}$ (resulting in hypoxia) and Ca, in the region close to it, which in turn is an impediment to the efficiency of radiotherapy and chemotherapy. This decrease is the largest in sites adjacent to the tumor and smaller away from the tumor. Such change in the concentrations of these elements is vital in the mechanism of cancer therapies. Starting from a fully occupied lattice, we introduce a distance-dependent removal probability q(d). The value of q(d) is 1 at and next to the tumor (center) and decreases linearly away from it to a limiting value $q_{p}$, which is the value of *q* at the lattice boundaries. We investigate the system properties as a function of $q_{p}$ and observe a significant decrease in the critical percolation threshold $p_{c}$ as $q_{p}$ decreases, falling from the standard value of $p_{c}=0.5927$ to approximately $p_{c}=0.20$. Furthermore, we demonstrate that the size of the spanning cluster and the total number of clusters exhibit a strong dependence on $q_{p}$ as well.

## 1. Introduction and the Model

The percolation model [1] is a classical paradigm in Statistical Physics for describing a binary disordered system, and together with the Ising model its constitutes the simplest model [2] for randomly mixed systems. While by their very nature these systems are mathematical/geometrical structures, nevertheless, in the past fifty years the rapid progress in the development of new algorithms has opened the way to a large variety of applications, ranging from randomly mixed crystals [3,4] to fluid flow in porous media [5,6], gelation and polymerization [7,8], porosity and adsorption [9,10], and more recently networks [11,12,13], studying their robustness [14], the spreading of diseases in epidemics [15,16], the spreading of rumors and gossip on social media [17,18,19,20,21], the spreading of crises and innovations in economic networks [22], and a large variety of other fields not possible to enumerate here. Recently [23,24], a crude percolation model was proposed by us to study the depletion of key chemical elements in the vicinity of cancerous tumor cells, which results in hampering the effectiveness of crucial therapies for the curing of cancer. That model presented preliminary findings on how the percolation phase transition changes with the depletion of these elements. Near and around the area of a tumor there is the so-called microenvironment (TME), an area that is strongly affected by the tumor and is characterized by a change in the normal density of constituents, producing a density gradient and degrading the results of applied therapies, such as chemotherapy and radiation. The center of mass of the tumor containing the tumor cells can then proceed more easily to metastasis, which is very precarious for the living organism. Specifically, in the TME the tumor is able to affect the oxygen content, which results in the depletion of $O_{2}$ or hypoxia [25,26,27,28], the concentration of extracellular potassium ions, leading to excess of $K^{+}$ or hyperkalemia [29,30,31], and its acidity, i.e., increase in $H^{+}$ or lowering of pH [32,33,34].

It is therefore important to explore in detail the constituents and concentration of the above species in the TME, its mass distribution, and its gradient density, as they all have important implications for understanding the tumor biology and the subsequent medical treatment. The important factor therefore is the gradient density of these constituents, as a function of distance from the center of the tumor. Thus, chemotherapy treatment may be effective only at the TME periphery and beyond because the drug cannot easily progress into the tumor center, as it may be hampered by the TME’s acidity. Similarly due to hypoxia, the $O_{2}$ depletion at the center of a tumor will differ from that at its periphery. Such depletion, while always reducing the efficiency of any radiation therapy, may also affect its combination with chemotherapy. Additionally, the distribution of the TME’s hyperkalemia may affect the success of immunochemistry.

In the current work we propose to study the interplay of the concentration gradient between the tumor core and the TME periphery using the percolation model. Notably, the information on the distributions of the molecular species discussed above may elucidate the importance of the different therapy options, i.e., whether radiation or chemotherapy is appropriate, or a combination therapy of these two, or even if surgery would be the most appropriate route. Imaging is also used to visualize, detect, diagnose, and monitor tumors within the body, and together with therapy its is often affected by tumor penetration difficulty, either by the drug or by the imaging contrast agent [35].

The percolation system is defined as a geometrical structure made of two components that are randomly mixed. In the case of a lattice, we consider the lattice sites as being either empty or occupied. Similarly, an analogous situation is if the bonds connecting pairs of sites exist or not. One could correspond the presence or absence of lattice sites with that of the molecular species discussed above. We use the probability *p* to designate if a site is occupied or not, with *p* being in the range 0<p<1. Thus, p=0 signifies a totally empty lattice, while p=1 corresponds to a fully occupied lattice. Occupied sites that are adjacent (nearest neighbors) form clusters of sites. Initially the lattice is empty (p=0). Sites start to become occupied when *p* increases, and clusters of sites are formed. The basic characteristic is that the system undergoes a sudden phase transition characterized by a sharp change in the connectivity of the system that depends entirely on the probability *p*. This transition is characterized by a critical value of the parameter *p*, which is called $p_{c}$, and it is defined as the point where for the first time a large spanning cluster is formed with occupied sites at opposite ends of the lattice. Such a transition is a second-order phase transition, and it is characterized by several properties, as indicated by a series of critical exponents that have been investigated in detail in the past [36]. In this context, the probability *p* acts as the control parameter of the system. The order parameter is defined as the probability P∞ that a site belongs to the spanning cluster, which determines the state of the system. In the classical percolation model, all sites are characterized by the same probability *p* of being occupied, uniformly distributed across the entire lattice. In the present work we define a new model in which *p* (the probability for a site to be occupied) is not uniform in the entire lattice but depends on the distance from a particular lattice point, with this point typically being the center of the lattice. This is done so in our effort to imitate the tumor microenvironment, for which we want a concentration gradient with respect to the central point. Our motive emanates from our effort to build a model in which a central point plays a key role in allowing neighboring sites to be occupied or not, depending on their distance from it. A real situation can be visualized in a tumor system in which the tumor itself is able to modify the concentration of key elements in its close neighborhood, such as $O_{2}$ and $K^{+}$, by reacting with them and depleting their needed concentration for the therapy to be effective in killing the tumor cells.

In the current study we start with a fully occupied lattice and then use an inverse percolation model. We start to randomly remove sites, but now instead of using the probability *p*, which is the same for the entire lattice, we define *q* as the probability that a given site will be removed from the lattice, which now depends on the distance from a certain point. We choose the center of the lattice as the “tumor cell” and we set q=1 at the lattice center. The removal probability *q* decreases linearly with the distance from the center, reaching a boundary value denoted by the parameter $q_{p}$ at the perimeter of the lattice.

## 2. Methodology

We start with a two-dimensional square lattice of size L×L. Initially, the lattice is fully occupied (p=1). Our goal is to remove sites, one by one, until we reach the critical percolation threshold, defined as the first moment when the largest connected cluster ceases to exist upon the removal of the last site. This point is commonly known as the red-bond point. In the classical site percolation (where sites are removed at random), the critical threshold is well-known, $p_{c}\approx 0.5927$, and is defined as the point where a connected cluster spans the lattice from one side to the opposite (top to bottom or right to left).

In this new model, a spatial bias is introduced in the site removal process. Sites are chosen at random to be inspected for possible removal. The probability of removal, *q*, is no longer uniform but depends on the site’s distance from the center of the lattice. The model is governed by the parameter $q_{p}\in \left[0,1\right]$, which represents the boundary value of the removal probability at the lattice perimeter. Specifically, the removal probability q(d) is a function of the distance *d* from the center such that q=1 at the origin and scales linearly to $q\left(d_{max}\right)=q_{p}$ at the boundary.

The probability of removal q(d) at a site located at a distance *d* from the lattice center is calculated using the linear gradient

(1) $$q\left(d,q_{p}\right)=1-\left(1-q_{p}\right)\frac{d}{d_{\max}}=1-\frac{2(1-q_{p})}{L-1}d$$

where $d_{\max}=\left(L-1\right)/2$ is the distance from the center to the perimeter of the lattice, *d* is the distance from the center to the site, and $q_{p}$ is the boundary parameter.

To further understand the dynamics described by Equation (1), it is instructive to analyze the extreme conditions of the function $q(d,q_{p})$ and their corresponding physical meanings. In the limit where $q_{p}=1$, the removal probability becomes constant across the entire lattice, q(d,1)=1 for all *d*, representing a scenario where the infection is so aggressive that it leads to a uniform and instantaneous removal of all sites. Conversely, the case $q_{p}=0$ represents the maximum possible spatial gradient; here, removal is certain at the center (q=1) but the probability vanishes at the perimeter (q=0), modeling a localized tumor where the infection’s impact diminishes completely at the boundaries. Furthermore, for any value of $q_{p}$, the center of the lattice (d=0) remains the primary source of the process with q=1, while at the perimeter ($d=d_{\max}$), the probability is identically $q_{p}$, confirming that $q_{p}$ acts as a control parameter that adjusts the intensity of the infection’s spatial decay. The distance *d* between a point (x,y) and the center $\left(x_{c},y_{c}\right)$ of the square lattice can be measured in several ways, and the choice of metric may impact the geometry of the iso-probability contours. These include the following ways: (1) the maximum norm metric ($L_{\infty }$), (2) the Euclidean distance ($L_{2}$ norm), and (3) the Manhattan distance ($L_{1}$ metric or taxicab geometry) [37]. For completeness we will briefly describe each one of them and schematically show their properties

(1) Maximum norm metric ($L_{\infty }$)

(2) $$d=\max(|x-x_{c}|,|y-y_{c}|)$$

The geometric locus of the iso-probabilities (points with the same *q*) forms a square.

(2) Euclidean distance ($L_{2}$ norm)

(3) $$d=\sqrt{{\left(x-x_{c}\right)}^{2}+{\left(y-y_{c}\right)}^{2}}$$

The iso-probabilities form circles.

(3) Manhattan distance ($L_{1}$ metric)

(4) $$d=|x-x_{c}|+|y-y_{c}|$$

The iso-probabilities form a diamond shape (square rotated by 45 degrees).

**Figure 1:**
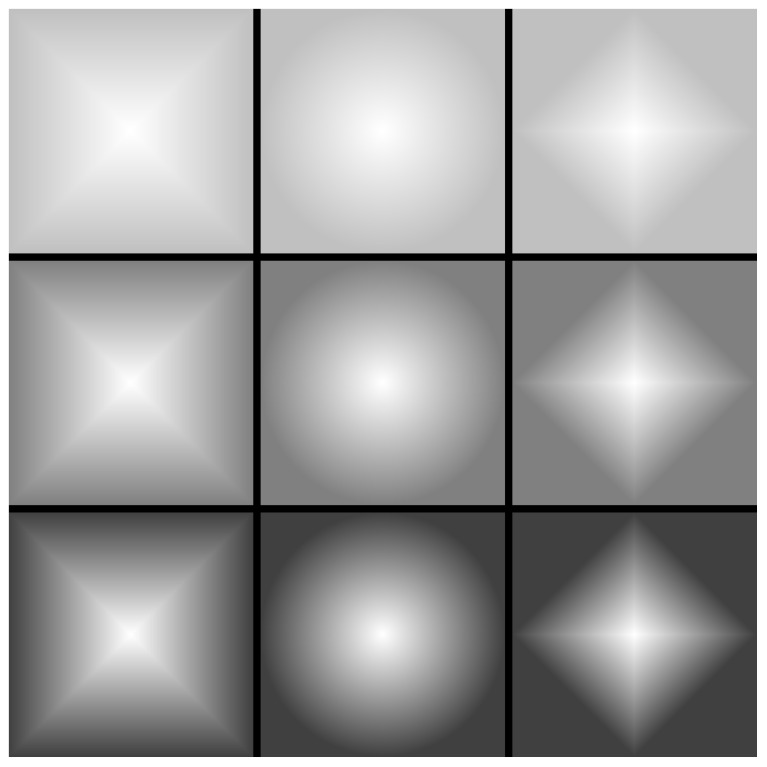
Iso-probability contours for site removal, showing the influence of distance metrics and perimeter removal probability ($q_{p}$). The columns represent the square ($L_{\infty }$), Euclidean ($L_{2}$), and Manhattan ($L_{1}$) distance metrics (**left** to **right**). The rows show decreasing probability $q_{p}$, with values $q_{p}=0.75$ (**top**), $q_{p}=0.50$ (**middle**), and $q_{p}=0.275$ (**bottom**). The color scale indicates the local probability of a site remaining in the lattice, defined as $1-q(d,q_{p})$. Specifically, darker regions (near the edge) correspond to a higher probability of remaining (1−q→1), while lighter regions (**center**) correspond to a higher probability of being removed (q→1). Here, $q_{p}$ denotes the value of the removal probability exactly at the perimeter of the lattice.

The spatial distribution of the removal probability and the resulting geometry of the lattice are illustrated in Figure 1. This figure showcases how the choice of distance metric ($L_{\infty }$, $L_{2}$, and $L_{1}$) reshapes the iso-probability contours, effectively altering the ’footprint’ of the infection. In the first column ($L_{\infty }$), the contours maintain a square symmetry, whereas the Euclidean metric ($L_{2}$) produces circular patterns, and the Manhattan metric ($L_{1}$) results in diamond-shaped contours. Moving from the top to the bottom rows, we observe the effect of decreasing the boundary parameter $q_{p}$. As $q_{p}$ is reduced, the gradient between the center (q=1) and the perimeter becomes steeper. Visually, the central lighter regions represent areas with a high probability of site removal (q→1), while the darker peripheral regions indicate a higher probability of sites remaining occupied (1−q)→1. This visualization confirms that for lower $q_{p}$ values, the ’survival probability’ of the cells increases significantly as we move toward the lattice edges, effectively confining the most intense part of the removal process to the core of the structure.

The choice of metric defines the shape of the region with the same removal probability. In the current work we will use the maximum norm metric (L∞ metric), although we do not expect significant differences using any of the other two ways.

The $L_{\infty }$ metric was chosen as the primary focus because its square iso-probability contours align perfectly with the boundaries of the square lattice used in our simulations. This alignment allows for a more straightforward interpretation of the boundary parameter $q_{p}$ and its effect on the global connectivity of the system.

By varying $q_{p}$, we effectively modulate the boundary conditions of the infection process. This parameter determines the steepness of the probability gradient between the center and the perimeter. Specifically, lower values of $q_{p}$ correspond to a more pronounced spatial decay of the removal probability, thereby increasing the likelihood of site survival as we move toward the lattice boundaries.

The simulation proceeds as follows: Initially, a specific value for the boundary parameter $q_{p}$ is assigned for the entire duration of the realization. In each step, a lattice location (i,j) is randomly chosen. If the site is already empty, a new location is chosen. If the site is occupied, its corresponding removal probability q(i,j) is calculated based on its distance *d* from the center, using the fixed value of $q_{p}$ in Equation (1). A uniformly distributed random number R∈[0,1] is generated and compared with the probability q(i,j). If R<q(i,j), the site is removed (becomes empty); otherwise, the site remains occupied. The process continues with the next randomly selected location. Following every successful site removal, the lattice is tested for the existence of the percolating cluster (spanning connectivity). This process continues until the red-bond point is found for the given realization.

In order to avoid checking to see if the spanning cluster has broken down after each site removal, which is very time-consuming, a two-stage method is used. First, a Coarse Search Phase is conducted where sites are removed in groups until non-percolation is detected. The Hoshen–Kopelman [1,2,4] algorithm is used effectively for fast cluster identification and percolation detection. Following the loss of spanning connectivity, a Fine Search Phase (Binary Search) is applied to the interval of the last removed sites. This binary approach precisely locates the minimum number of sites that must be restored (or, conversely, the maximum number that can be removed) to maintain connectivity, thus accurately defining the critical threshold for the specific $q_{p}$ and realization. To ensure statistical convergence and minimize fluctuations, we perform N=1000 independent realizations for each set of parameters and average the resulting values. Exactly at the critical point, various quantities are calculated, such as the critical concentration $\langle p_{c}\rangle$, the size of the maximum cluster $\langle S_{\max}\rangle$, and various cluster moments, in order to fully characterize the percolation transition under the influence of the spatial probability gradient.

## 3. Results

**Figure 2:**
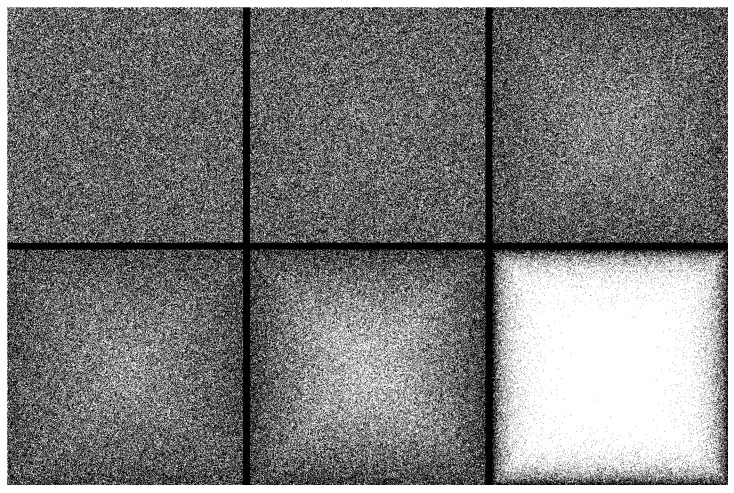
Site occupancy on an L×L square lattice at the critical probability $\langle p_{c}\rangle$ for different perimeter probabilities $q_{p}$ ($q_{p}=1,0.8,0.5,0.4,0.2,0.01$). Unoccupied sites are shown in white, and occupied sites are shown in black. Note that for smaller $q_{p}$ values, the higher density of black sites near the perimeter is consistent with the higher survival probability (1−q) shown in the darker regions of Figure 1.

We first vary the $q_{p}$ value, and we calculate the value of the critical percolation threshold point. In Figure 2 we show schematics of typical realizations of the lattice for several different $q_{p}$ values.

**Figure 3:**
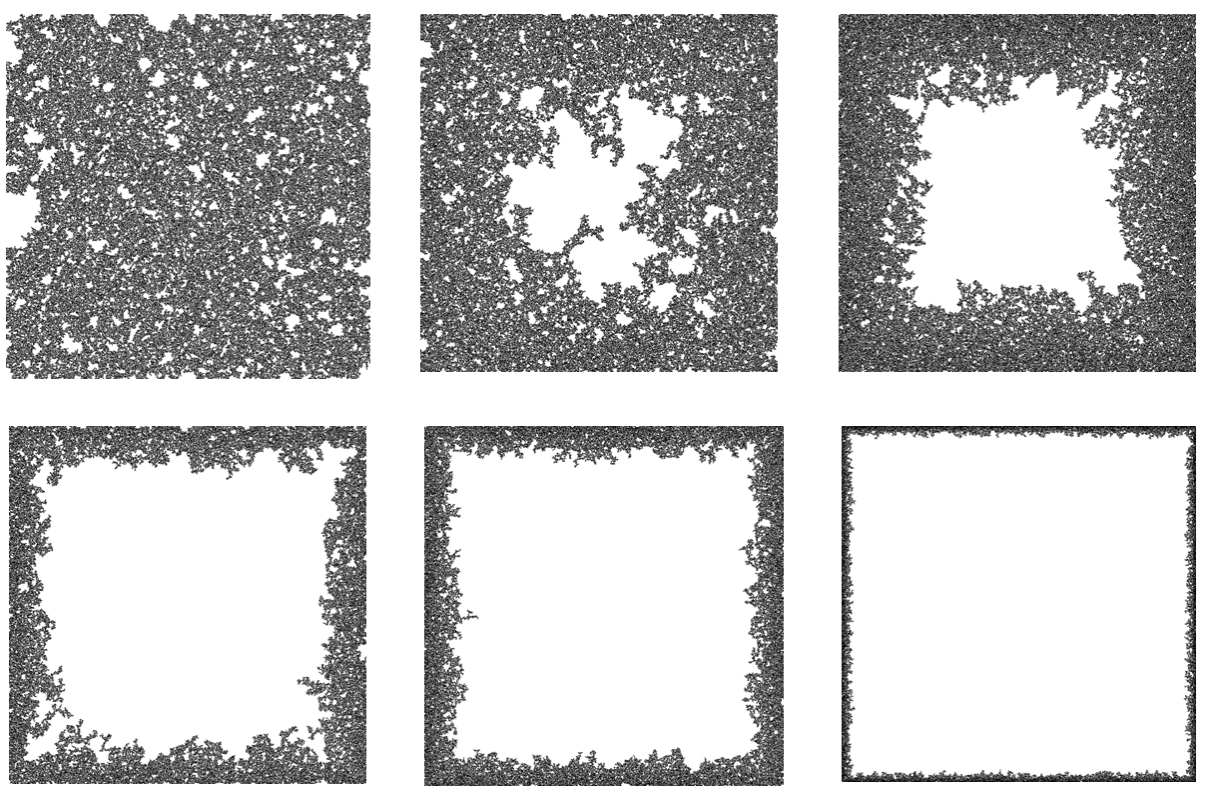
Giant cluster formed at $\langle p_{c}\rangle$ for the same cases as Figure 2. The black pixels represent the sites belonging to the spanning cluster. The concentration of the cluster toward the perimeter for decreasing $q_{p}$ reflects the spatial gradient defined in Equation (1).

The top-left panel of Figure 2, representing $q_{p}=1$, corresponds to the classical percolation, which results in the well-known value of $p_{c}=0.5927$. As $q_{p}$ is reduced we see in the several plots of Figure 2 that the lattice starts to become empty in the middle, while the area close to the perimeter is still relatively more occupied, as is expected in this model. The corresponding spanning clusters for the same $q_{p}$ values are given in Figure 3, where only the largest cluster is shown, verifying the results of the previous figure.

**Figure 4:**
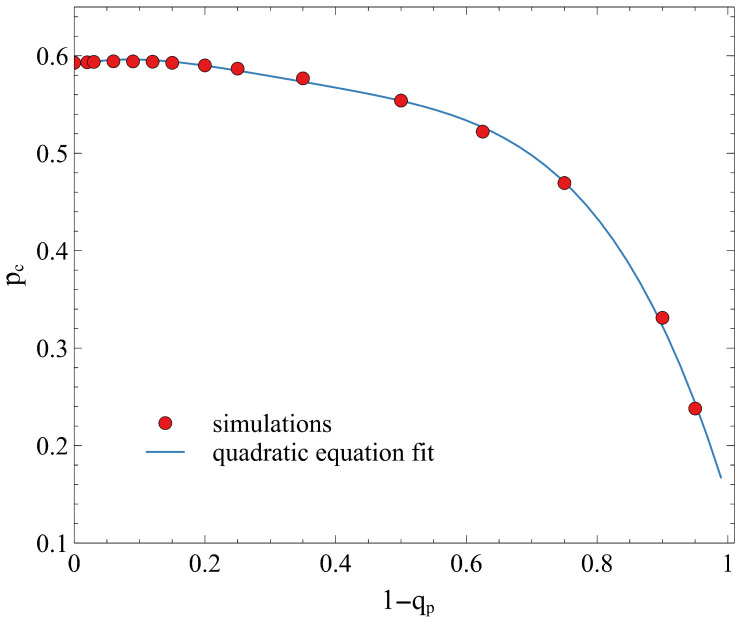
Dependence of the critical percolation threshold $p_{c}$ on the perimeter parameter $\left(1-q_{p}\right)$ for the case of the $L_{\infty }$ metric. The solid line represents a fourth-order polynomial fit of the form $p_{c}\left(q_{p}\right)=\sum_{i=0}^{4}a_{i}{\left(1-q_{p}\right)}^{i}$. The best-fit coefficients are determined as $a_{4}=-1.7327$, $a_{3}=2.3102$, $a_{2}=-1.1787$, $a_{1}=0.1555$, and $a_{0}=0.5902$. The reduced chi-square value ($\chi_{red}^{2}=0.9707$) indicates an excellent agreement between the proposed model and the numerical data, supporting the geometric interpretation of the transition. Simulations were for lattice sizes L=1001 and 1000 iterations.

The actual value of $p_{c}$ is plotted in Figure 4 as a function of $q_{p}$. Here we actually plot the value of $\left(1-q_{p}\right)$, which corresponds to the non-removal probability, so that at the value $1-q_{p}=0$ we recover the $p_{c}$ value of $p_{c}=0.5927$. We observe a small decrease in the $p_{c}$ value in the range up to $1-q_{p}=0.5$ and a much sharper decrease in the range 0.5–1.0 in a parabolic-like curve. This suggests that the presence of a highly protected perimeter ($q_{p}\to 0$) allows the percolating cluster to survive even at lower overall occupation probabilities. In the extreme case where $q_{p}=1$, the removal probability q(d,1) becomes uniform throughout the entire lattice. Under this condition, the spatial gradient vanishes, and every site is removed with certainty, leading to a completely vacant lattice. This represents the absolute upper bound of the infection process, where the system cannot sustain any connectivity. While we tried several fittings for the points of Figure 4, the reduction in $p_{c}$ follows a non-linear trend that is accurately captured by a fourth-order polynomial fit, as detailed in Figure 4.

**Figure 5:**
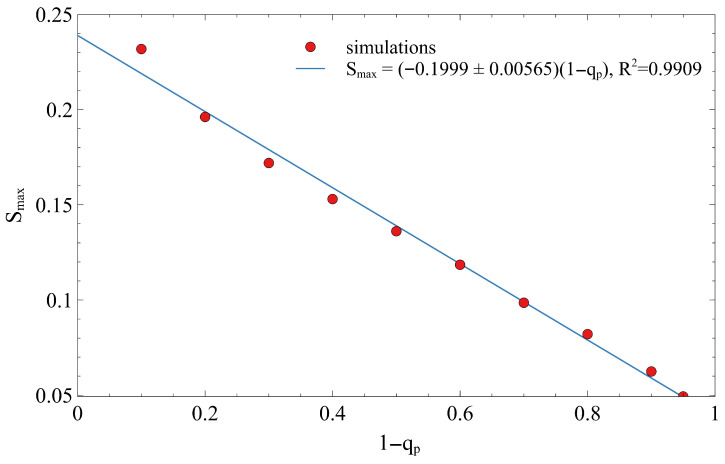
The variation in the maximum cluster size ($S_{\max}$) as a function of the non-removal probability at the perimeter ($1-q_{p}$) for a square lattice of size L=1001. The relationship is linear $R^{2}=0.9909$, exhibiting a slope of approximately −0.2.

We next calculate the relative size of the largest connected cluster, $S_{\max}$ (normalized by the total number of sites), at the critical threshold $p_{c}$. Figure 5 illustrates that the maximum cluster size, $S_{\max}$, decreases linearly as a function of $\left(1-q_{p}\right)$. The line has a negative slope (approximately −0.2), indicating a clear linear relationship. This linear dependence, described by the relation $S_{\max}\approx -0.20\left(1-q_{p}\right)$, suggests that as the perimeter sites become less likely to be removed (higher $1-q_{p}$), the relative size of the largest connected cluster at the critical point decreases. The spatial inhomogeneity forces the spanning cluster to highly constrained regions closer to the center ($q_{p}=1$), effectively reducing the maximum cluster size that the lattice can support before fragmentation.

**Figure 6:**
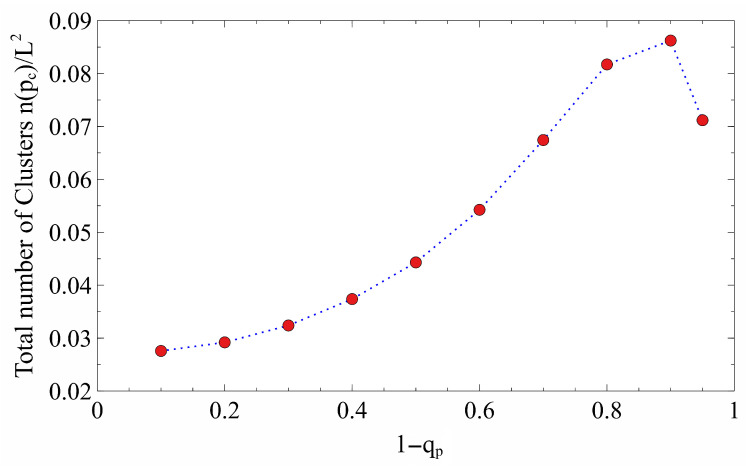
The behavior of the normalized mean number of total clusters, $n\left(p_{c}\right)/L^{2}$, at the critical point for various values of the perimeter non-removal probability ($1-q_{p}$) for a square lattice size of L=1001. The mean total number of clusters exhibits a quadratic increase with respect to the perimeter non-removal probability ($1-q_{p}$).The red circles represent the simulation data, while the dashed blue line is added as optical guide.

The total number of non-percolating clusters (normalized by the total number of sites), $n\left(p_{c}\right)/L^{2}$, was then calculated at the critical point $p_{c}$ for each $q_{p}$ value. Figure 6 shows that $n(p_{c})$ increases as $\left(1-q_{p}\right)$ increases, reaching a maximum near $(1-q_{p})\approx 0.9$, and then falls sharply at higher values. We observe a parabolic-like behavior for most of the $1-q_{p}$ range. The subsequent sharp decrease is consistent with the rapid drop in $p_{c}$ observed in Figure 4. The overall increase in the number of clusters indicates that the gradient in *q* promotes fragmentation and the creation of numerous small, isolated clusters at the critical point, especially when the perimeter is highly protected (low $q_{p}$).

**Figure 7:**
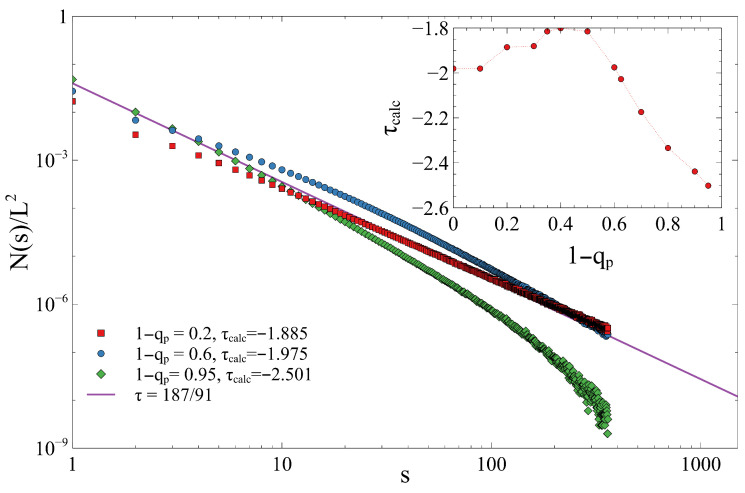
Normalized cluster size frequency distribution ($N\left(s\right)/L^{2}$) versus the cluster size (*s*) on a log–log scale, examining the effect of the perimeter non-removal probability ($1-q_{p}$) on cluster statistics at the critical point. The inset shows the change in the slope $\tau_{\mathrm{calc}}$ of the cluster size distributions with respect to $1-q_{p}$. The red circles represent the simulation data, while the dashed line is added as optical guide.

The distribution of cluster sizes, N(s) (the number of clusters of size *s*), is crucial for characterizing the universality class of the transition, as it typically follows a power law: $N\left(s\right)∼s^{-\tau }$. Figure 7 (in a log–log plot) confirms that the cluster size distribution follows a power law for all tested values of $q_{p}$. The calculated exponent, $\tau_{\mathrm{calc}}$, varies significantly with the parameter $1-q_{p}$. For the classical 2D random percolation, the exponent is τ=187/91≈2.055. The inset of Figure 7 shows that $\tau_{\mathrm{calc}}$ starts close to the theoretical value for $1-q_{p}\to 0$, reaches a minimum near $1-q_{p}\approx 0.5$, and then increases sharply for large $1-q_{p}$, indicating a significant deviation from the classical percolation universality class due to the *q*-gradient. This suggests that the spatial inhomogeneity introduced by the gradient is strong enough to change the effective critical exponents, implying that the gradient percolation model may belong to a different universality class than random percolation.

## 4. Discussion and Conclusions

In the present work we introduced a percolation model with new rules for the occupancy of the sites. This research is motivated by the necessity to provide a quantitative framework to explain the concentrations of molecular ingredients in the vicinity of the tumor microenvironment. The binary nature of the percolation model could represent the existence or absence of key molecules as a function of the distance to the tumor cells. While the classical percolation model has been successful in the past in describing several realistic systems, in recent years several variations of the model have appeared in the literature, which may describe different applications. For example, see the Achlioptas model [38]. In all these variations the key characteristic is that the critical parameter values vary significantly from the classical model. This may lead one to prepare a system that exhibits a desired critical threshold value [39]. In the present case we show how the depletion zone around the center of the lattice strongly affects the creation of the spanning cluster, reducing the critical threshold to a very low value. This reduction was shown to follow a parabolic behavior. The parameter $q_{p}$ serves to regulate the spatial gradient of the removal probability between the lattice center and the perimeter, thereby controlling the boundary conditions of the infection process. We find that the size of the maxi-cluster falls linearly with $1-q_{p}$ with a slope of ≈−0.20. Our results may be useful in explaining the behavior of the tumor microenvironment, and subsequently the control of the efficiency of possible therapies, in an effort to find the optimal amount of radiation and chemotherapy tailored for each individual case.

The proposed model’s ability to simulate spatial gradients via the parameter $q_{p}$ aligns with established findings on the heterogeneity of the tumor microenvironment (TME) [25,40]. Such gradients are known to impede the delivery of therapeutic agents and oxygen, particularly in hypoxic regions [41,42], which can now be quantitatively analyzed through the lens of distance-dependent percolation.

## Data Availability

The data and code that support the findings of this study are openly available in GitHub at https://github.com/lef27064/PercWithDistDepData, accessed on 18 December 2025.
